# Syndrome de Melkersson-Rosenthal

**DOI:** 10.11604/pamj.2015.22.228.7887

**Published:** 2015-11-11

**Authors:** Madiha Mahfoudhi, Khaled Khamassi

**Affiliations:** 1 Service de Médecine Interne A, Hôpital Charles Nicolle, Tunis, Tunisie; 2 Service ORL Hôpital Charles Nicolle, Tunis, Tunisie

**Keywords:** Syndrome de Melkersson-Rosenthal, oedème labial, paralysie faciale périphérique, Melkersson-Rosenthal syndrome, labial edema, peripheral facial palsy

## Image en medicine

Le syndrome de Melkersson-Rosenthal est une entité rare. Dans sa forme complète, il se caractérise par un œdème cutanéo-muqueux de la face, une paralysie faciale périphérique récidivante et une langue plicaturée. Il peut poser un problème diagnostique dans sa forme incomplète. Son traitement est essentiellement médical, parfois chirurgical. Femme âgée de 58 ans, diabétique et hypertendue, a consulté pour une hémiparésie faciale gauche d'installation brutale, évoluant depuis 5 jours sans signes auditifs ou vestibulaires associés. L'examen physique a objectivé une paralysie faciale périphérique gauche (grade V de House), un œdème des lèvres et une langue plicaturée. L'examen biologique était sans anomalies. L'audiométrie tonale était normale. Plusieurs diagnostics étaient évoqués en particulier un lymphome ou une sarcoïdose. L'examen anatomopathologique (biopsie labiale) a révélé un œdème du chorion associé à un infiltrat lympho-plasmocytaire. Le diagnostic du syndrome de Melkersson-Rosenthal a été retenu. Le traitement s'est basé sur une corticothérapie par voie orale et une rééducation motrice. L'Evolution était marquée par une amélioration de la paralysie faciale périphérique gauche (grade II de House) et une disparition de l’œdème labial.

**Figure 1:**
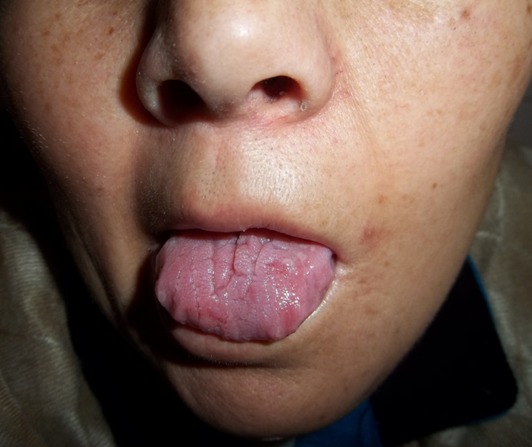
Langue plicaturée

